# Mistaken Impressions on Nitrous Oxyd

**Published:** 1871-03

**Authors:** 


### Editorial.

### MISTAKEN IMPRESSIONS ON NITROUS OXYD.

In the January number of *The Cincinnati Lancet and Observer*, the leading communication is from the pen of W. W. Dawson, M. D., of Cincinnati. It is lengthy, interesting, and shows commendable research. In the main we like it, but must call attention to an obvious error in its teaching, which we the more cheerfully do, as Dr. D. and the writer are natives of the same hazel thicket—a most delectable place, where the trees grow a little taller, the sun shines brighter, and the birds sing clearer than elsewhere.

In speaking of NITROUS OXYD, Dr. D. says: "Its safety depends on the fact that persons are kept under its influence but for a moment. Protract its administration as we do chloroform and ether, and its victims would far outnumber those of all the anæsthetic agents combined. No surgeon or physician who has stood by and witnessed a dentist give nitrous oxyd for the extraction of teeth, would be willing to hazard any individual under the full influence of this gas, for an operation that would last *one half minute.* The appearance of the person while inhaling, as he is pushed beyond the point of excitement, to a condition of insensibility, is fearful; the pulse is quick, the breathing labored, the vessels of the face and neck are turgid, the face assumes an ashy hue; indeed, the whole aspect is one of danger."

The above is a graphic picture of a series of rather formidable symptoms, and probably falls even short of the reality, as seen by Dr. D.; but as we have spent more months than he has minutes in the investigation of nitrous oxyd, it will not be considered presumptuous if we "show unto him a more excellent way."

For nearly three-quarters of a century, experiments with nitrous oxyd, were conducted mainly by adventurous mountebanks and self-styled "professors," who administered to the patients their own breaths and something else, the composition of which was unknown alike by professors and victims. Even the experiments of Sir Humphrey Davy are almost valueless, from the fact that in all of them the breath was re-inhaled with the gas. Even as lately as the fall of 1866, the "Colton Dental Association" practiced this method, giving the gas from rubber bags; and not far from this time, Mr. Colton, in an article published in one of the dental journals, advocated the re-inhalation of the breath, as necessary to complete anæsthesia. As far as we know, this mode of administration was then in very general use.

With such administration, the symptoms detailed by Dr. D. will be often observed; but it is *carbonic acid*, and not nitrous oxyd, that causes them. And it has been a view from this standpoint, that induced Dr. Richardson to make the remark quoted by Dr. D.; for it is true from no other. Richardson's remark is as follows: "It can not be too widely understood, that protoxyd of nitrogen, is not an *anæsthetic*, in the true sense of the word, but an *asphyxiating agent; that its effects are identical with those of poisoning by carbonic acid gas.*"

It is not likely that a greater amount of error could be embraced in so short a sentence, as the oxyd *is an anæsthetic*, and will *revive an asphyxiated patient more promptly than atmospheric air.*

But Dr. D., or some one, may say that these symptoms have been seen where valved inhalers have been used. This is true; and carbonic acid is the agent producing them still. During the first few inhalations of nitrous oxyd, carbonic acid is given off in great abundance, so as to almost smother the patient when proper precautions are not observed. The exhaling valve is nearly always far too small; and when the expiration is retarded in this, or any other way, much of the carbonic acid passes back into the circulation. A disposition to economize gas, and a belief (from erroneous teachings by those who should have known better), that atmospheric air must not be admitted to dilute the gas in the air cells, result in the poisoning of many patients by the return of carbonic acid to the blood, in obedience to the law of gaseous diffusion. In this way the symptoms may be almost the same as if the gas were administered in connection with the breath.

But if the operator will bear these facts in mind, and will admit fresh air, as freely as wanted, whenever the breathing is in the least disturbed, it will be but a short time till the patient will prefer the gas to the atmosphere, and his breathing will be perfectly tranquil, and in a few minutes he will be wide awake, even while breathing nothing but the nitrous oxyd ; and through all there will be nothing like asphyxia or suffocation, and the heart's action is changed only by mental emotion, if changed at all. It has been breathed many times for fifteen or twenty minutes, without any change in the pulse.

We have administered the gas almost daily, and often many times a day, for months, without seeing the complexion darkened, the face or neck turgid, or the muscles rigid. In a majority of the cases the patients were tranquil throughout, not delirious, and often completely anæsthetized, while perfectly conscious.

If, however, sufficient attention is not given to the expiration, and carbonic acid is allowed to pass over, there will be muscular contractions and delirium, with darkening of the complexion, in short, the formidable array described by Dr. D.

It is the practice of many to prop the mouth open with a cork, or a wooden block, before administering the gas for extraction ; and, if the patient is to be drugged as above, with carbonic acid, this precaution is, perhaps, necessary, as the flexors will be generally found rigidly contracted ; but in the light of science, the practice is both barbarous and unnecessary. When pure gas has been properly administered, the under jaw will be perfectly manageable. We never prop the mouth open, and find no occasion to do so.

But this is too long. Again we repeat, *if the complexion has been darkened, impure gas has been used, or the patient has been smothered.* W.

